# Race, College Graduation, and Time of Retirement in the United States: A Thirty-Year Longitudinal Cohort of Middle-Aged and Older Adults

**DOI:** 10.31586/ojer.2024.1029

**Published:** 2024-08-16

**Authors:** Shervin Assari, Amanda Sonnega, Hossein Zare

**Affiliations:** 1 Department of Internal Medicine, Charles R. Drew University of Medicine and Science, Los Angeles, CA, United States; 2 Department of Family Medicine, Charles R. Drew University of Medicine and Science, Los Angeles, CA, United States; 3 Department of Urban Public Health, Charles R. Drew University of Medicine and Science, Los Angeles, CA, United States; 4 Marginalization-Related Diminished Returns (MDRs) Center, Los Angeles, CA, United States; 5 Institute for Social Research, University of Michigan, Ann Arbor, MI, United States; 6 Department of Health Policy and Management, Johns Hopkins Bloomberg School of Public Health, Baltimore, MD, United States; 7 School of Business, University of Maryland Global Campus (UMGC), College Park, United States

**Keywords:** Education, Social Determinants, Retirement, Aging, Population Groups

## Abstract

**Introduction:**

College education is typically associated with the ability to work in less physically demanding occupations, allowing for a later retirement age. However, research indicates that highly educated Black individuals often work in more demanding occupations, which affects their retirement age.

**Aim:**

Building on the Minorities’ Diminished Returns (MDRs) literature, we tested whether the benefit of college education on delaying the time of retirement is weaker for Black compared to White middle-aged and older adults.

**Methods:**

We utilized data from the Health and Retirement Study (HRS), which includes a 30-year longitudinal follow-up of a nationally representative sample of middle-aged and older adults in the United States. Education levels at baseline were categorized as less than college graduate (some high school, GED, high school diploma, or some college) and college graduate. The outcome was the time to retirement, measured from wave 2 to wave 15 (baseline to 30 years later). We graphed survival curves and used independent samples t-tests to assess associations between college graduation and time of retirement, overall and by race.

**Results:**

Our analysis included 6,803 White and Black participants who were employed at baseline and followed for up to 30 years. Overall, there was a positive association between college graduation and retirement timing, with individuals with higher education retiring later. However, we found significant racial differences in the retirement age of college graduates, indicating notable racial disparities in the effects of college graduation on retirement timing, disadvantaging Black college-educated individuals. Specifically, among Whites, but not Blacks, college education was associated with later retirement.

**Conclusion:**

Consistent with Minorities’ Diminished Returns theory, the positive effect of college education on retirement timing are weaker for Black than for White middle-aged and older Americans. To address racial disparities, it is insufficient to focus solely on economic disparities. While closing the educational gap is important, we must also work to equalize labor market experiences for Black and White individuals with similar educational credentials. Structural factors contributing to the diminished returns of college education for Black populations must be addressed to effectively close racial disparities.

## 1. Introduction

Education tends to sort individuals into positions with higher pay, more benefits, better promotion opportunities, and less physical demand, which in turn leads to better economic and health outcomes [95]. Those with higher education levels often work in more favorable occupations, such as white-collar jobs, and typically experience less stress [96]. As a result, considering that a significant portion of human life is spent in work, individuals with higher education tend to maintain better health throughout their careers [1]. Conversely, those in manual labor jobs face high stress and physical demands, which can result in greater wear on their health [3]. Given the impact of occupation type on well-being [2], it is crucial to equalize occupational experiences across racial groups at similar educational levels. Unfortunately, even with equivalent education, Black individuals are more likely to occupy jobs with higher physical and mental wear and tear, even when they have similar educational attainment [93].

Given the history of slavery followed by segregation and social stratification, the effects of education and occupation on economic and health outcomes are influenced by race [94]. Considering the complex, non-linear, and non-additive links between education and race on economic status [4], it is essential to compare Black and White people regarding the effects of education on work experiences and life trajectories. Due to Jim Crow laws and redlining, the U.S. experienced significant segregation of Black and White communities, contributing to divergent educational and occupational trajectories for Black and White individuals. This has influenced many aspects, including the returns on education and occupation [5]. Understanding the disparities in life conditions among racially diverse groups with similar education or occupations helps us comprehend how systemic racism, labor market discrimination, and social stratification shape the living condition differences of Black and White people with comparable credentials [6]. Results from these studies will reveal the intricate interplay between education/occupation and race in the U.S. [7].

As shown by Minorities’ Diminished Returns (MDRs) [8], the effects of educational attainment and occupation on economic status, as well as health and well-being, tend to be weaker for Black individuals compared to White individuals [9, 10, 11, 12, 13]. While several studies have shown that economic and health outcomes are worse for Black people than White people at the same educational levels (racial disparities persist across each education level) [14, 15, 16, 17], few studies have compared the diminished returns of education and occupation for Black middle-aged and older adults in the United States [18, 19, 20].

Some occupational classes, such as managerial, professional/specialty, sales, and clerical admin, are associated with lower stress and manual labor and may experience less wear and tear [21]. In contrast, occupations in service and operator roles exhibit noticeable difficulty [22]. There are more Black individuals in service and operator occupations, while White individuals are more represented in managerial, professional, and clerical/admin positions [23]. This labor market division is partly due to the lower education levels of Black people; however, a significant portion of this division results from the lower quality of education and labor market discrimination and segregation, directing Black people to service and manual labor and White people to more prestigious, lower-stress, higher-paying jobs [24]. Consequently, there is less representation of Black people with the same education in managerial, professional/specialty, and clerical admin positions, with a higher prevalence in service and operator roles, even at the same education levels [25]. This race-based occupational stratification has broader health implications, as the nature of work in these categories can contribute to different stressors and wear-and-tear on the body [26].

Systemic barriers include discriminatory hiring practices and limited access to career advancement opportunities [27]. These barriers contribute to the overrepresentation of Black individuals in service and operator positions, which often involve manual labor and are associated with higher levels of physical and psychological stress [28]. Despite the underrepresentation of Black individuals in professional/specialty and managerial roles [29], there remains a question: if Black and White individuals end up in such low-stress, higher-pay jobs, would we still see diminished health returns from occupational attainment for Black people compared to White people [18, 19, 20]?

Manual jobs, such as those in service and operator categories, are characterized by elevated stressors and wear-and-tear due to the physical demands of the work [30, 31]. This differential recruitment to various occupational classes, driven by systemic factors, may contribute to observed health disparities by race [32]. The overrepresentation of Black individuals in manual jobs may expose them to heightened health risks, potentially explaining part of the health disparities seen across racial lines. Recognizing and addressing these occupational disparities is crucial for advancing health equity, ensuring fair access to opportunities across job classes, and fostering environments where individuals of all races can thrive [33].

Labor market discrimination persists as a challenge [34, 35, 36], leading to stark occupational disparities between Black and White individuals, even when educational qualifications are comparable [37]. Deep-seated biases within hiring processes and workplace structures result in Black individuals facing barriers that funnel them into lower-status, less remunerative occupations [38]. This discrimination occurs at various stages, from hiring decisions to promotions, perpetuating racial wage gaps and socioeconomic inequalities [39, 40, 41]. Beyond economic disparities, occupational segregation reinforces social stratification, limiting access to resources and opportunities for career progression. Addressing labor market discrimination is crucial for dismantling these occupational disparities and fostering a more equitable and inclusive workforce based on merit rather than discriminatory practices [42, 43].

## 2. Aims

This study seeks to explore the complex interplay between race and college graduation on the timing of retirement in middle-aged and older adults. Building on the Minorities’ Diminished Returns (MDRs) framework, we argue that race, as a historical and sociological construct, significantly impacts retirement age beyond educational attainment, primarily through occupational class disparities. Black and White individuals with college degrees may not have equal access to well-paying, low-stress occupational opportunities due to historical and contemporary societal structures that perpetuate health and economic disparities by race.

We will utilize longitudinal data from the Health and Retirement Study (HRS) to investigate whether college graduation differentially influences the age of retirement for Black and White middle-aged and older individuals. This study aims to extend the existing knowledge on MDRs and elucidate why health and economic disparities persist between Black and White individuals across education levels into older age. Given that racial disparities in occupational prestige persist even among those with similar educational backgrounds, our results may have implications for dismantling these disparities in health and economic well-being over the life course, particularly for middle-aged and older adults.

It is imperative for US policymakers to develop targeted interventions and policies that address the unique needs of highly educated minority populations, promote health equity beyond education, and foster the well-being of highly educated middle-aged and older Black adults, who represent a growing segment of the United States population.

In this study, we examine the combined influences of college graduation (education) and race on the timing of retirement in a nationally representative sample of middle-aged and older adults in the United States. Utilizing 30 years of follow-up data from the Health and Retirement Study (HRS), our study may reveal new mechanisms by which college graduation yields weaker economic and health outcomes for Black populations compared to White populations, particularly in later stages of life.

## 3. Methods

### 3.1. Design and Setting

This study utilized data from all available biennial waves of the Health and Retirement Study (HRS) spanning from 1992 to 2020. The HRS gathers comprehensive data on a variety of participant characteristics, including demographic, socioeconomic, social, psychological, economic, employment, and health aspects, in addition to health behaviors and health service utilization. It also collects extensive information related to retirement, including the timing of retirement. Data collection was conducted through telephone or face-to-face interviews, with proxy interviews used when participants were unavailable. For this analysis, the publicly released RAND HRS Longitudinal File, updated in March 2023, was utilized.

### 3.2. Sample and Sampling

The HRS employs a cohort-based longitudinal design, with the initial cohort recruited in 1992. Participants aged 51 to 61 at the baseline in 1992 were recruited using a national area probability sample. For the current study, only the core (primary) sample recruited in 1992 was included to provide the longest follow-up period. The participants in this study were born between 1931 and 1941, representing middle-aged and older adults aged 51-61 living in US households in 1992.

### 3.3. Analytical Sample

The analytical sample comprised US adults aged 51-61 in 1992 who identified as either White or Black. Individuals from other racial groups were excluded from the analysis. All participants from the HRS core sample were eligible for analysis regardless of follow-up duration or time of mortality, except those who identified as retired at baseline. Therefore, the analytical sample consisted of 6803 Black or White non-retired working middle-aged or older participants at baseline, followed for up to 30 years.

### 3.4. Measures

#### 3.4.1. Time of Retirement (Outcome).

The primary outcome of this study was the age at the time of retirement. This was treated as a time-to-event variable, consisting of a binary event variable (0 = not retired, 1 = retired) and time to event (number of years followed until retirement occurred). Retirement was defined as a participant who reported working in a previous wave and subsequently reported not working or being retired in a following wave. The timing of retirement was measured biennially.

#### 3.4.2. Educational Attainment (Predictor).

Educational attainment was measured in five levels: less than high school, high school graduate, GED, some college, and college graduate. Given the previous research that shows college graduation generates fewer health and economic benefits for Black than White Americans, we treated education as a binary dichotomous variable, with less than college and college graduate as the levels. Lower education was the reference category. Educational attainment was self-reported at baseline in 1992.

#### 3.4.3. Baseline Age (Control Variable).

Baseline age was treated as a continuous variable, calculated based on the number of years since birth, and measured in 1992.

#### 3.4.4. Gender (Control Variable)

Gender was treated as a dichotomous variable, coded as 0 for female and 1 for male, and measured in 1992.

#### 3.4.5. Marital Status (Control Variable).

Participants reported their marital status at baseline. A dichotomous variable was used to indicate being married (coded as 1) versus any other status (coded as 0) in 1992.

### 3.5. Data Analysis

Data analysis was conducted using SPSS version 25.0 (IBM Corporation, Armonk, NY, USA). For univariate analyses, means (with standard deviations [SD]) and absolute/relative frequencies (n and %) were reported. Multicollinearity among the study variables was assessed and ruled out prior to running the models. For comparative analysis, we used Survival curves and Cox regression models for analysis. We also used independent t test and Analysis of Variance (ANOVA) for comparison of average time to retire. Results were reported as mean time to retire as well as standard deviation.

### 3.6. Ethics Statement

The Health and Retirement Study (HRS) protocol received approval from the University of Michigan Institutional Review Board. All participants provided written informed consent. The data collection, storage, management, and analysis were conducted in a fully anonymous manner. Since this study utilized fully de-identified, publicly available data, it was classified as non-human subject research according to the NIH definition.

## 4. Results

**Figure 1.**
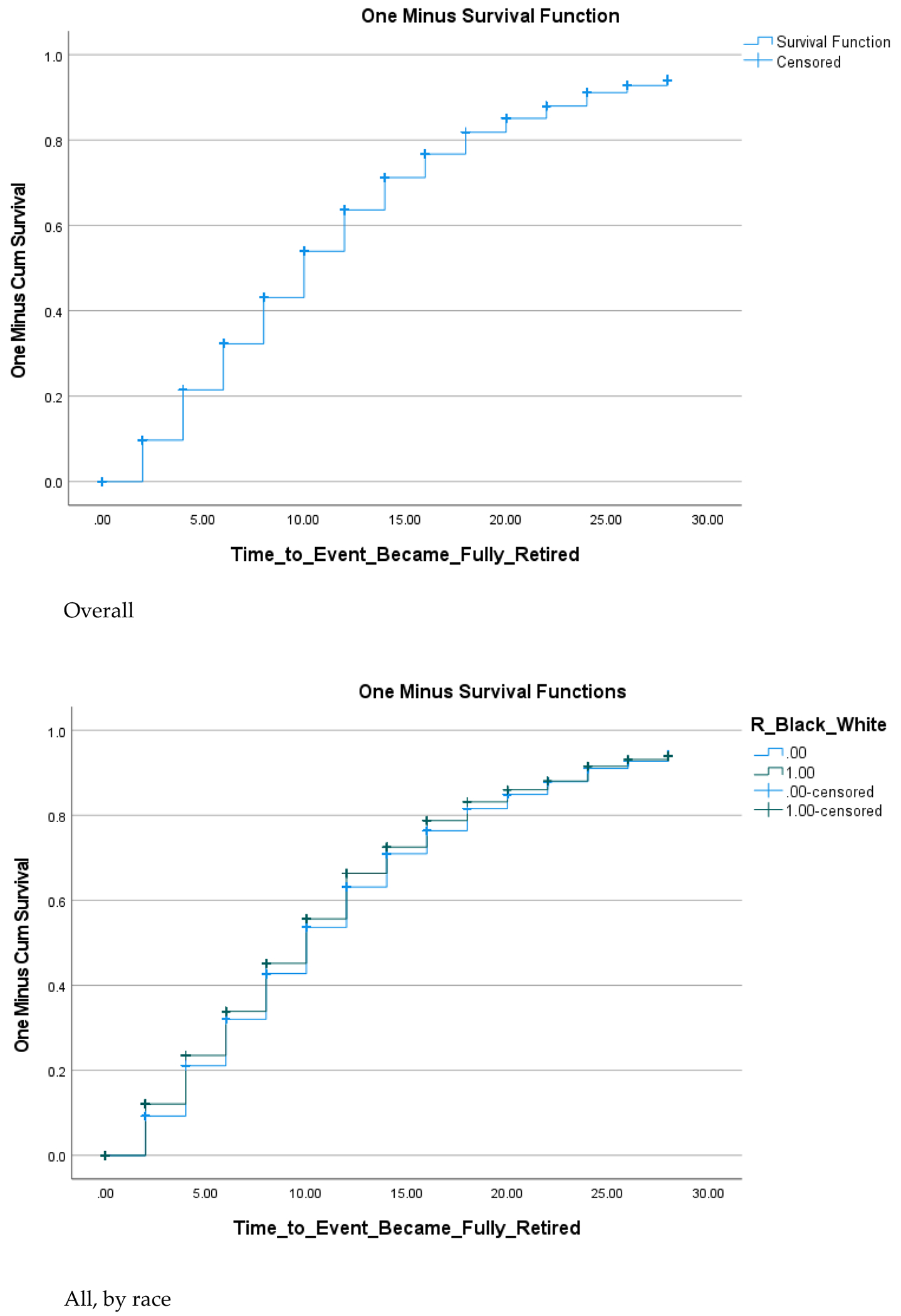

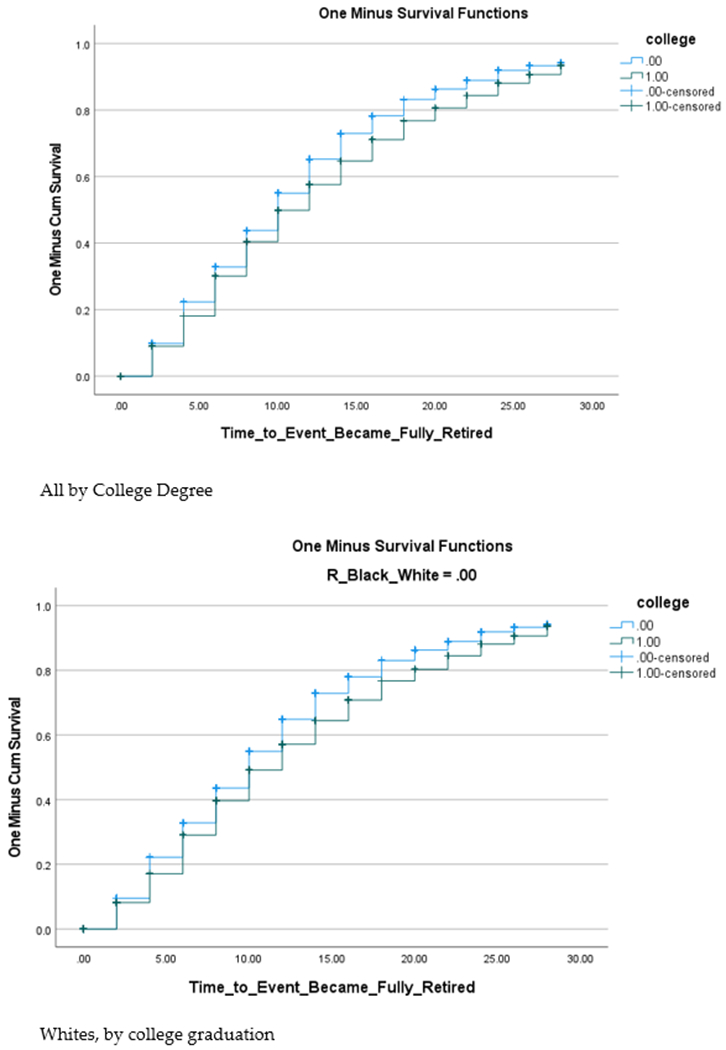

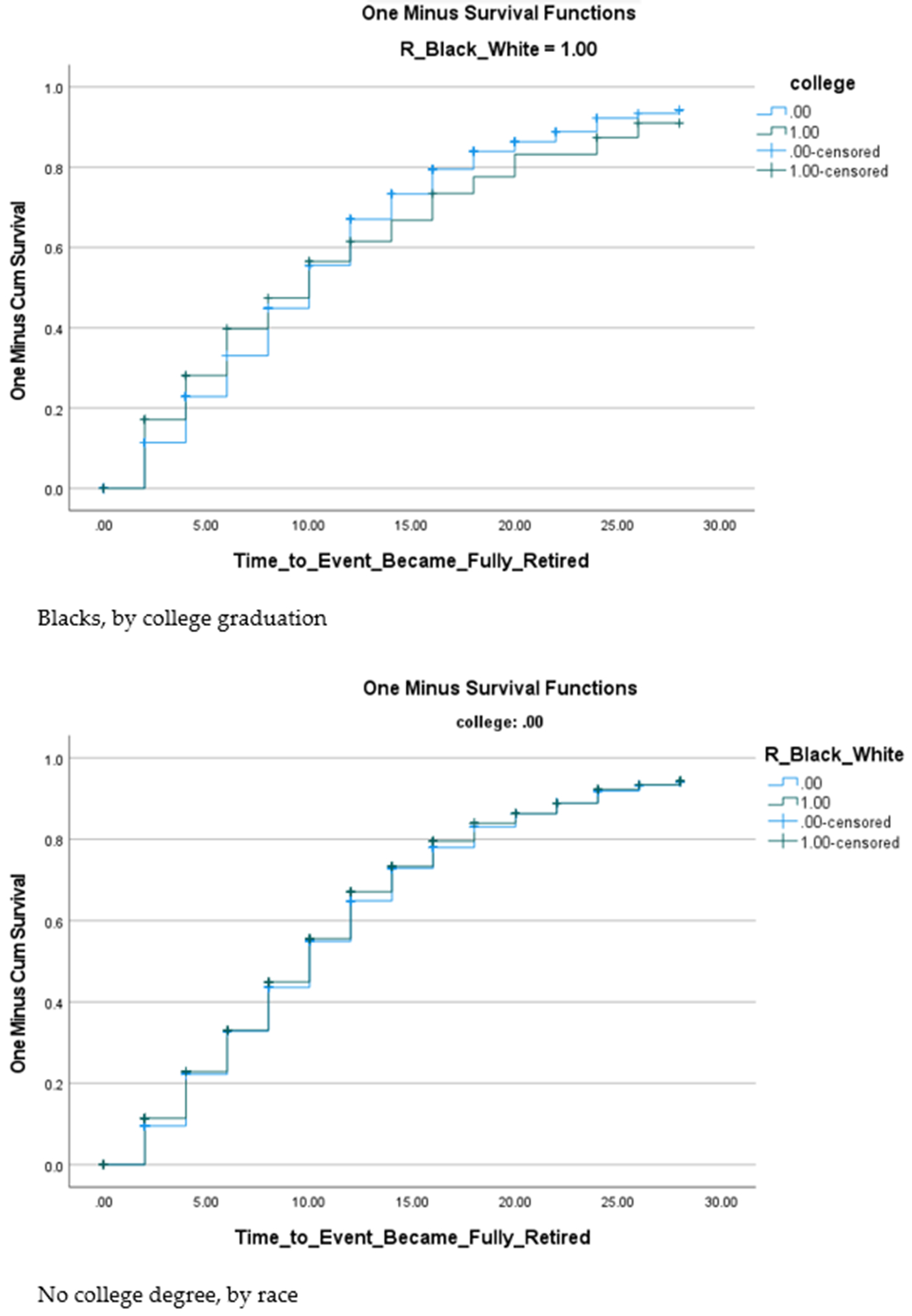

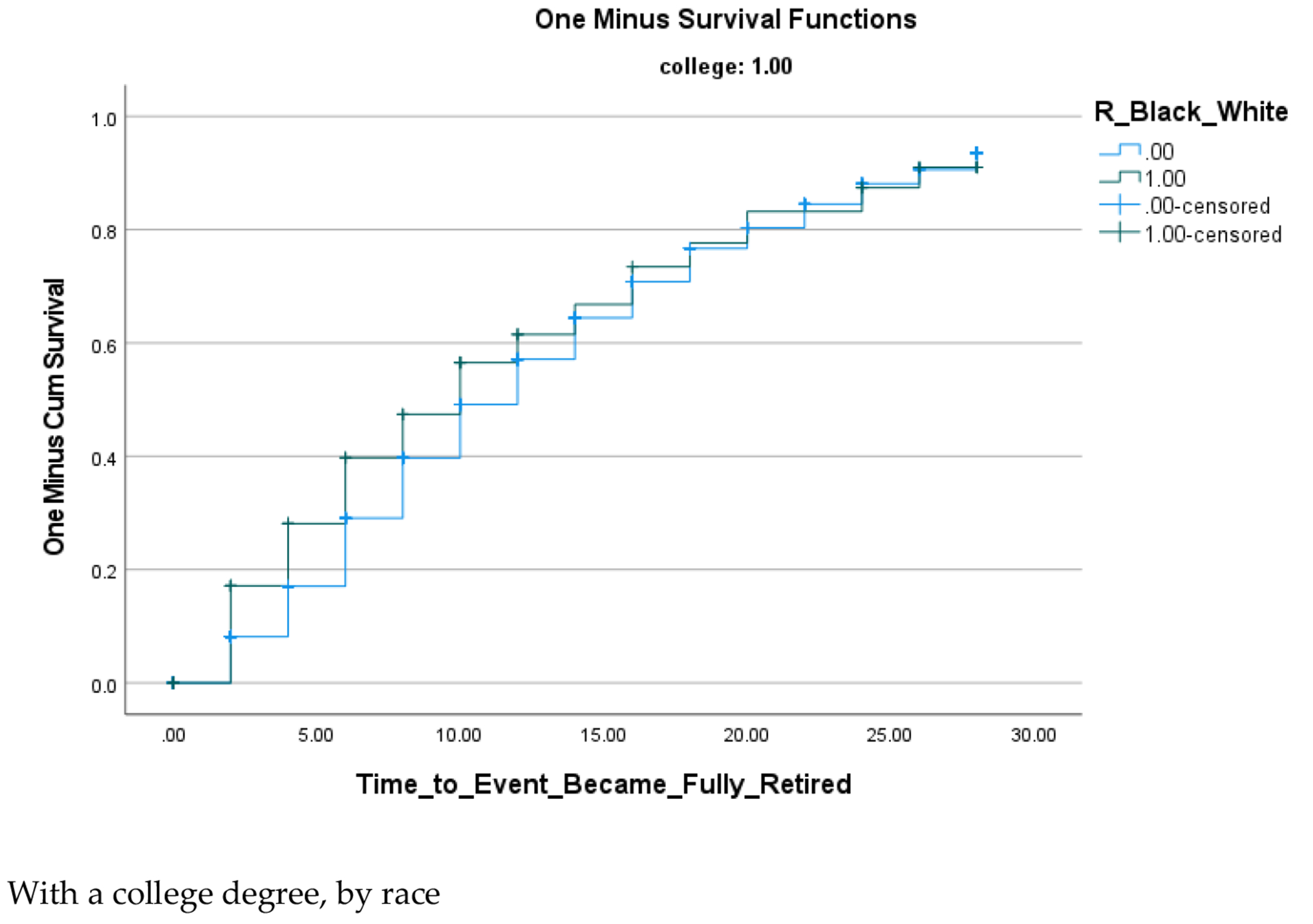
Time to Retire Over the Course of Follow Up (30 Years) by Race and College Graduation

Table 1 provides descriptive data for the entire sample as well as by race. The analysis included 6,803 participants who were followed for up to 30 years. Among these participants, 16.7% were Black and 83.3% were White. The data showed that Black participants were less likely to be college graduates compared to White participants and tended to retire earlier than their White counterparts (Figure 1).

As indicated in Table 2, higher educational attainment was associated with a later age of retirement over the 30-year follow-up period. Similarly, Whites had an average later retirement age compared to Black individuals. Among people with low education, there was no significant racial difference in the average time to retirement. However, among those with a college degree, race was associated with the time to retirement, with highly educated Black individuals retiring earlier than highly educated White individuals.

## 5. Discussion

In this analysis of 30-year follow-up data from the Health and Retirement Study, which is a nationally representative sample of middle-aged and older Americans, there was a positive association between college graduation and time/age of retirement. However, this effect was significant for White but not Black individuals compared to White individuals.

Our first observation aligns with the role of SES indicators such as high education in enhancing economic wellbeing [1, 67, 68]. Several mechanisms may explain why people with high education can retire later and stay in the labor market longer, including working in better jobs with lower levels of wear and tear and reduced exposure to stressors [69, 70, 71, 72]. College education allows people to work in less stressful, more manageable jobs that tend to offer superior benefits, such as improved healthcare accessibility, better working conditions, and the maintenance of individual health trajectories [73]. In this way, better occupations, which allow people to retire later, connect college education to economic prosperity and health [74]. These effects may be also in part because people who are college graduated work in jobs that allow that

Our second finding supports past research on Minorities’ Diminished Returns (MDRs) [8], which posits that the economic and health advantages derived from resources like higher education are systematically weaker for Blacks compared to Whites. This theory highlights the persistence of racial health and economic disparities beyond educational credentials, driven by structural factors and racism that shape how populations utilize resources such as education to achieve better occupations [75, 76]. Attributed to racism, social stratification, and segregation, the Minorities’ Diminished Returns theory suggests that education’s contribution is least pronounced for groups facing racialization, discrimination, and blocked opportunities, even when high educational attainment is secured [9, 77].

Few studies have tested the MDRs of college graduation in middle-aged and older adults [78, 79, 80]. These limited studies suggest that MDRs can be seen in the effects of occupational classes on the health of Black middle-aged and older people in the United States [81]. In a related study within the HRS, we observed the effects of occupational classes such as professional and managerial roles on several objective and subjective mental and physical health outcomes. However, there were Black-White disparities in the impact of professional occupational class on health outcomes, with this positive effect being weaker for Black individuals compared to White individuals [97].

Historical slavery and ongoing racism, combined with segregation and social stratification, have resulted in a drastic difference in the daily experiences of Black and White people for centuries, which continues today [82]. This historical and contemporary marginalization of Black communities has blocked not only access but also the returns of resources and opportunities for Black people [83]. These lasting effects result in worse education, worse occupations, lower pay, and fewer benefits for similarly educated Black and White people [84]. Systemic discrimination has created structural barriers that increase the cost of social mobility for Black people [85]. Recognizing and addressing these deeply rooted historical, structural, and environmental challenges is essential for achieving social and economic justice for Black Americans [86, 87, 88].

### 5.1. Implications

The implications of our findings extend to the realms of research, policy, and practice. There is a need for targeted initiatives that address disproportionate barriers in the lives of Black people across education and occupational classes. Policymakers should develop tailored policies, programs, and interventions specifically designed to dismantle structural and institutional racism, focusing on removing barriers hindering the professional advancement of Black individuals. There might be social, environmental, and ecological barriers and challenges unique to highly educated Black people. Improving the employability of highly educated Black people and reducing labor market discrimination in Black communities and predominantly Black labor markets are crucial components of an inclusive strategy. Institutions at the government, state, and local levels should invest more in combating discriminatory practices within the labor market and educational systems, with policies crafted to account for the diminished impacts of high-prestige occupations, such as those within the professional class, on Black populations. It is imperative that endeavors to enhance occupational outcomes translate into tangible improvements in health outcomes for all, fostering a more equitable and inclusive society.

### 5.2. Limitations

This study is not without limitations. All data were self-reported, which is prone to measurement bias and differential validity and psychometric properties based on race. Furthermore, the dataset was limited to the original HRS cohort, consisting of middle-aged and older adults who were 51 to 61 years old in 1992. As such, the results may not be generalizable to all middle-aged and older adults from other birth cohorts. There is also a chance that this study may have biases due to unmeasured confounders and omitted variables. We dichotomized education into two categories: less than college and college graduates, which is a coarse operationalization of education. Our study solely incorporated individual-level data, lacking the inclusion of area-level variables. Therefore, the effects of broader contextual factors on the observed returns of education could not be studied.

## 6. Conclusion

There are Minorities’ Diminished Returns in the effects of college graduation on time/age of retirement in middle-aged and older adults. We need to address racial disparities that are not explained by education and may reflect labor market discrimination and lower quality of discrimination, in part due to segregation.

## Figures and Tables

**Table 1.** Descriptive Data Overall and by Race and College Graduation (n = 6,803)

	All		White		Black			College −		College +		
	n	%	n	%	n	%		n	%	N	%	
Gender*												
Female	3417	50.2	2763	48.7	654	57.7	< 0.001	2887	52.2	530	41.7	< 0.001
Male	3386	49.8	2906	51.3	480	42.3		2644	47.8	742	58.3	
Race*												
White	5669	83.3						4526	81.8	1143	89.9	< 0.001
Black	1134	16.7						1005	18.2	129	10.1	
College Degree *												
Not	5531	81.3	4526	79.8	1005	88.6	< 0.001					
Yes	1272	18.7	1143	20.2	129	11.4						

* p<0.05

**Table 2.** Average Time Before Retirement in Middle-Aged and Older Adult Americans Based on Race and College Graduation

Group	N	Mean	Std. Deviation
Race x Education			
White College −	3826	9.21	6.71
Black College −	788	8.74	6.62
White College +	1030	10.71	7.34
Black College +	109	9.45	7.72
Race			
White	4856	9.53	6.88
Black	897	8.82	6.77
Education			
College −	4614	9.13	6.70
College +	1139	10.59	7.38
All	5753	9.42	6.86

## Data Availability

HRS data are publicly available here: https://hrsdata.isr.umich.edu/data-products/rand.
